# Health and Wealth in America

**DOI:** 10.3389/ijph.2024.1607224

**Published:** 2024-03-15

**Authors:** William B. Weeks, Juan M. Lavista Ferres, James N. Weinstein

**Affiliations:** ^1^ AI for Good Lab, Microsoft Corporation, Redmond, WA, United States; ^2^ Microsoft Research, Microsoft Corporation, Redmond, WA, United States

**Keywords:** public health, social determinansts of health, economic activity, wealth, income

## Introduction

Health and wealth has a long-established relationship, and recent studies found that local economic improvements are associated with improved population health measures [1, 2]. Building on this research, wealth redistribution has been proposed as a way to extend United States longevity and narrow the US-OECD average mortality gap [3].

To address health inequities, public policy has focused on understanding the social determinants of health, defined by the World Health Organization as “the non-medical factors that influence health outcomes” and “the conditions in which people are born, grow, work, live, and age and the wider set of forces and systems shaping the conditions of daily life.” Many studies found associations between exposure to worse social determinants conditions and worse health outcomes.

As an example, food insecurity is associated with premature mortality and lower life expectancy in the United States [4]. However, a recent randomized controlled study found that an intensive food-as-medicine program–one designed to address food insecurity and that provided 10 healthy meals per week for an entire household as well as dietitian consultations, nurse evaluations, health coaching, and diabetes education–increased engagement with preventive healthcare and improved self-reported diet in the intervention group but did not improve glycemic control relative to the control group [5].

While both of these studies found that the less wealth and resources a person has, the shorter their lives tend to be, suggested ways to resolve the issue vary. These papers’ editorialists suggest that “only broad policy solutions, expanding beyond equalizing food security and wealth, respectively, are needed to address the disparities in longevity. Thus, multi-pronged policy solutions, aimed at optimizing multiple social determinants of health will be required to make sustainable improvements in longevity that equitably span multiple population subgroups.” [6] The authors of the food-as-medicine program think that “future research that tests how [food-as-medicine] program parameters are related to health improvements may inform the optimal design of food-as-medicine programs.” [5] While the food insecurity authors advocate for “a higher prioritization in tackling food insecurity as a means to improve population health and reduce health inequities,” they note that a study limitation is that food insecurity might simply be a marker for poverty [4].

What if most social determinants of health are merely flags for low wealth and income? Might that allow for a more streamlined approach to reducing health inequities by addressing the fundamental causes of them: inequitable wealth distribution? Considering this how might the United States develop a long-term strategy to enhance equitable access to economic resources and opportunities, with an expected outcome being improved population health?

## A Role for a Federal Health Authority

We have previously suggested the establishment of a Federal Health Authority (FHA). Fashioned after the Federal Reserve, the FHA would have five mandates: to improve population health while reducing health inequities, to coordinate efforts to mitigate health crises, to supervise and regulate health entities, to ensure consumer protection, and to align with national research institutions to monitor the population’s health and identify health threats [7]. Coordination with the Federal Reserve would be critical to a successful FHA because of the critical interplay between health and local economic conditions. Just as the Federal Reserve acts independently of political pressures to secure the safety of the United States economy, so the Federal Health Authority–potentially an evolution of the United States Department of Health and Human Services, one explicitly recognizing the interplay of health and wealth–would act independently to secure and improve the health of the workers supporting that economy. In concert with measures of financial performance–like gross national product *per capita*–the FHA would seek to improve health-related measures that contribute to financial performance, by reducing the number of people on social security disability, increasing the number of people who have no health conditions, or increasing the number of healthy years lived.

There are concerns that using tax laws alone to spur investment designed to improve local economic activity that may improve population health is an inadequate approach, one that may reduce income, but not wealth, gaps [8]. Although overt wealth redistribution may be a quicker and more effective way to improve health outcomes [3], it is an approach not likely to be embraced in America. And while leveraging tax laws may enrich the rich [8], we believe that careful coordination of economic and health policy that includes measurement of health outcomes associated with economic investment and, potentially, tying health outcomes to economic returns may be part of a successful approach that is pragmatic and might work within a capitalistic-democratic society, albeit more slowly that overt and sudden wealth redistribution, to achieve health and wealth equity.

However, a simpler, more palatable, and more achievable approach might be establishing a universal, unconditional, individual, periodic, cash payment based universal basic income, one that has no means testing (thereby reducing bureaucratic costs) that is distributed to every American: those with higher incomes would have the benefit taxed away. In high-, middle-, and low-income countries, such programs are effective in improving educational attainment, health risk factors, and health outcomes [9]. Potentially, offices that run current means-based programs (for example, the Supplemental Nutrition Assistance Program, social security disability payments, child tax credits) could be dismembered, providing additional resources for the universal basic income program.

## Why Corporate Leaders, Politicians, and the Population Might Be Interested

Within a capitalist society, the pursuit of profit drives many decisions. Incorporating health outcomes into measures of economic activity would have the impact of emphasizing long-term profitability over short-term profitability.

There are several reasons corporate leaders would want to pursue health-oriented economic prosperity. First, just as has happened with climate change, public pressure could encourage leaders to adopt policies and approaches that are more supportive of population health. Second, having a healthier workforce could increase productivity and reduce healthcare costs for corporate leaders, thereby improving short-term profitability. Finally, a healthier population that can afford to buy products throughout increasingly long lives will support longer-term sales revenue cycles, improving corporate fiscal sustainability.

Politicians might be interested because they could articulate the health benefits of economic programs that they put in place. Health benefits may be easier to explain, more tangible, and more motivating than complex economic policies. And the measurement of the impact of local economic policies on population health will help politicians describe their influence on that population.

Finally, the population would benefit. Despite longevity decreases attributable to “deaths of despair” in the 2010s and the COVID pandemic more recently, life expectancy has trended upward in the United States since the Civil War. But longevity in a nursing home is not a desirable state and healthy living into old age could generate a “third demographic dividend,” wherein people continue their societal contributions into old age [10].

## Conclusion

The strong relationship between health, income, and wealth in the United States suggests that all economic policy is health policy. While rapid wealth redistribution may synthetically address wealth-based longevity, it is an unpragmatic solution in a capitalist democracy. However, the development of a Federal Health Authority working in concert with the Federal Reserve to highlight and prioritize economic policies that generate positive health externalities could, over the longer term, address health and wealth inequities, develop a healthier workforce that continues to flourish well into old age, and contribute to long- and short-term corporate profitability.
